# Society News

**Published:** 1897-05

**Authors:** 


Society News. After the business session of the Lower Austrian Veterinary Society in Vienna, January 23d, the members accepted the invitation of Prof. Josef Bayer to visit the new clinic of the Veterinary Institute, where the following interesting cases had been prepared for demonstration: 1. Operation on a horse with radiating cancer, which had been under treatment for six months. 2. A plastic operation on the root of the front foot of a horse. 3. Cataract caused by artificial conditions, total on the left eye and nail-shaped on the right. 4. Congenital cataract on both eyes. 5. Huge melanotic tumor on the right cheek of a gray horse. 6. Breaking of the muscles on the under part of the right thigh. The demonstrations were received with great applause.—*Thierärztliches Centralblatt,* February 15, 1896.

